# Improved fertility following a gonadotropin-releasing hormone treatment on day 2 of an estradiol and progesterone-based timed-artificial insemination protocol in lactating dairy cows

**DOI:** 10.3168/jdsc.2022-0212

**Published:** 2022-03-31

**Authors:** Carlos E.C. Consentini, Tiago O. Carneiro, Humberto Neri, Emiliana O.S. Batista, Lucas O. e Silva, Alexandre H. Souza, Roberto Sartori

**Affiliations:** 1 Department of Animal Science, University of São Paulo, Piracicaba, SP, Brazil, 13418-900; 2 Bela Vista Farm, Tapiratiba, SP, Brazil, 13760-000; 3 Biotran Biotecnologia, Alfenas, MG, Brazil, 37132-346; 4 Adventist University Center of São Paulo, Engenheiro Coelho, São Paulo, Brazil, 13165-970; 5 Cargill Animal Nutrition and Health, Campinas, SP, Brazil, 13091-611

## Abstract

The present study evaluated the addition of gonadotropin-releasing hormone (GnRH) concomitant or 2 d after the beginning of protocols initiated with estradiol benzoate (EB). A total of 459 multiparous and 371 primiparous lactating Holstein cows were enrolled in the study. Weekly cohorts of cows were randomly assigned to 1 of 3 experimental groups that differed in the strategy to initiate the timed AI (TAI protocol. On d 0, all cows received a 1.55-g progesterone (P4) implant. Additionally, cows in the EBd0 group received 2 mg of EB i.m.; cows in the EBd0-GnRHd0 group were treated simultaneously on d 0 with 2 mg of EB plus 100 µg of gonadorelin diacetate tetrahydrate (GnRH) i.m.; and cows in the EBd0-GnRHd2 group received 2 mg of EB on d 0 and 100 µg of GnRH 48 h later (d 2). The remaining treatments in the protocol were similar among groups and included 0.53 mg (i.m.) of cloprostenol sodium (PGF_2α_) on d 7, followed by a second PGF_2α_ treatment on d 9 (at the time of P4 implant withdrawal) and 1 mg of estradiol cypionate i.m. Then, TAI was performed on d 11 (48 h after P4 removal) in all experimental groups. We detected an effect of treatment on pregnancy per AI (P/AI) on d 30, in which cows from the EBd0-GnRHd2 group demonstrated greater fertility than EBd0 cows, whereas cows in the EBd0-GnRHd0 group did not differ among EBd0 and EBd0-GnRHd0 (40.5 vs. 30.4 vs. 34.4%, respectively). In summary, GnRH treatment at the beginning of an estradiol and P4-based TAI protocol increased fertility only when GnRH was given on d 2. Moreover, a more pronounced positive effect of this strategy was observed in particular classes of cows: multiparous cows, cows with greater milk production, and those receiving the first service.

## Highlights

• GnRH on d 2 of timed AI (TAI) protocols initiated with estradiol benzoate increases fertility.
• Only estradiol benzoate on d 0 of TAI protocols decreases fertility of dairy cows.
• GnRH on d 2 of TAI protocol increased pregnancy per AI of multiparous and higher-producing cows.

## Graphical Abstract

**Figure:**
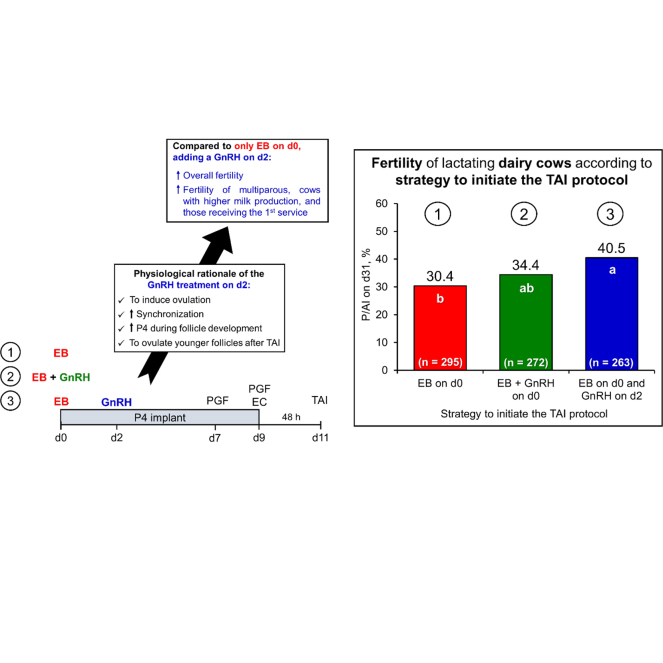
**Summary:** This study evaluated 3 strategies to initiate an estradiol/progesterone-based timed-artificial insemination protocol: (1) estradiol benzoate (EB) only on d 0, (2) EB plus GnRH on d 0, and (3) EB on d 0 and GnRH on d 2. Compared with the negative control group (EB only on d 0), adding GnRH on d 2 increased overall fertility, and particularly benefited the following groups of cows: multiparous cows, cows with higher milk production, and cows receiving the first postpartum service.

There are critical points during timed AI (**TAI**) programs that can optimize fertility of lactating dairy cows (Consentini et al., 2021). Initially, it is important to properly synchronize the emergence of a new follicular wave; this is essential to control the age of the ovulatory follicle (Monteiro et al., 2015). Moreover, the presence of a corpus luteum (**CL**) and high circulating progesterone (**P4**) concentrations during development of the preovulatory follicle are positively associated with pregnancy per AI (**P/AI**; Bisinotto et al., 2015; Melo et al., 2016).

Regarding synchronization of emergence of a new follicular wave, GnRH can be administered to induce ovulation, which is followed by emergence of a new follicular wave within 24 h, as commonly used in Ovsynch-type protocols (Pursley et al., 1995). In a recent compilation of studies by Borchardt et al. (2020), an overall ovulation incidence after GnRH treatment of 51.4% (2,204/4,291) was demonstrated. However, the ovulatory response varies among studies, being influenced by several physiological aspects such as presence of a CL (Borchardt et al., 2020), steroid hormone concentrations (Stevenson and Pulley, 2016), stage of estrous cycle (Vasconcelos et al., 1999), use of presynchronization protocols (Bello et al., 2006), and dose of GnRH (Giordano et al., 2013). Another often-used strategy to synchronize follicular emergence is causing atresia of the follicles in response to a combination of estradiol (**E2**) and P4, such as in E2/P4-based protocols (Bó et al., 1995; Barros et al., 2000). The circulating P4 profiles during the TAI protocol may differ according to the strategy used at the beginning. For instance, when GnRH causes ovulation, a new follicular wave initiates simultaneously with the development of a new CL throughout the protocol, and both factors are associated with greater P/AI (Giordano et al., 2013; Melo et al., 2016; Borchardt et al., 2020). In contrast, in E2/P4-based protocols, previous studies reported that approximately 25% of cows failed to have a new follicular wave emergence, and about 40% of cows underwent CL regression before the scheduled treatment with PGF_2α_. These events were associated with lower fertility in lactating dairy cows (Monteiro et al., 2015; Melo et al., 2016, 2018).

In a previous study, initiating the TAI protocol with GnRH instead of estradiol benzoate (**EB**) improved ovarian dynamics (CL presence at PGF_2α_), P4 milieu (higher P4 at PGF_2α_), and fertility in lactating dairy cows (Melo et al., 2016). A frequently implemented TAI protocol in commercial dairy herds initiates with EB and has an extended protocol length and longer proestrus, with the first PGF_2α_ on d 7, the second on d 9 (at P4 implant removal), and cows inseminated on d 11 (Pereira et al., 2015). Adding a GnRH at the beginning of this protocol increased fertility of lactating dairy cows (Pereira et al., 2015). However, GnRH given on d 2 could promote better fertility, because in cows ovulating after GnRH given on d 0, the ovulatory follicle may be too old or overexposed to LH due to protocol length and a longer proestrus.

Thus, the objective of the present study was to evaluate 3 strategies to initiate TAI protocols in lactating dairy cows: treatment with EB plus P4 implant only (**EBd0**) or additional treatments with GnRH, either simultaneously with the EB treatment on d 0 (**EBd0-GnRHd0**) or 2 d later (d 2; **EBd0-GnRHd2**). The main hypothesis was that inclusion of a GnRH treatment on d 0 or d 2 would increase P/AI of lactating dairy cows and that the GnRH on d 2 would promote greater fertility than GnRH on d 0.

Expecting an increase in P/AI ranging from 5 to 10 percentage points (e.g., 30% vs. 35 to 40%), a minimum sample size of 300 cows was determined after a power calculation using PROC POWER of SAS 9.4 (SAS Institute Inc.; power = 0.80 and α = 0.05). The experiment was conducted in 2 commercial dairy farms located in southeastern Brazil, both with 305-d average milk production of 9,000 kg. The Animal Research Ethics Committee of Luiz de Queiroz College of Agriculture of the University of São Paulo (ESALQ/USP) approved all procedures involving cows in this study (CEUA 5112290720). Farms had approximately 700 lactating Holstein cows milked thrice daily and fed twice with a TMR based on corn silage and a corn and soybean meal-based concentrate with minerals and vitamins balanced to meet or exceed the nutritional requirements of lactating dairy cows producing 40 kg/d of milk (NRC, 2001). All cows had ad libitum access to water and were housed in freestall barns bedded with sand and equipped with fans.

A total of 459 multiparous and 371 primiparous lactating Holstein cows were enrolled in the study from November 2015 to August 2016. Weekly cohorts of cows were randomly assigned according to parity and number of service (first postpartum TAI and resynchronization of ovulation protocols initiated at nonpregnant diagnosis 31 d after a prior AI), to 1 of 3 experimental groups that differed in strategy to initiate the TAI protocol (Figure 1). On d 0, all cows received a 1.55-g P4 implant (PRID Delta, Ceva); additionally, in the EBd0 group, cows received 2 mg of EB (Estrogin, Biofarm). Cows assigned to the EBd0-GnRHd0 group were treated simultaneously on d 0 with 2 mg of EB plus 100 µg of gonadorelin diacetate tetrahydrate (GnRH, Cystorelin, Merial) and, in the EBd0-GnRHd2 group, cows received 2 mg of EB on d 0 and 100 µg of GnRH 48 h later, on d 2. The remaining treatments in the protocol were similar among all groups, and included 0.53 mg of cloprostenol sodium (PGF_2α_, Veteglan, Hertape Calier) on d 7, followed by a second PGF_2α_ treatment on d 9 (at the time of implant removal) and 1 mg of estradiol cypionate (**EC**, Cipionato-HC, Hertape Calier). The TAI was performed on d 11 (48 h after P4 removal) with conventional Holstein semen in all experimental groups, and pregnancy diagnosis was performed by ultrasound examination 31 d after TAI.

**Figure 1:**
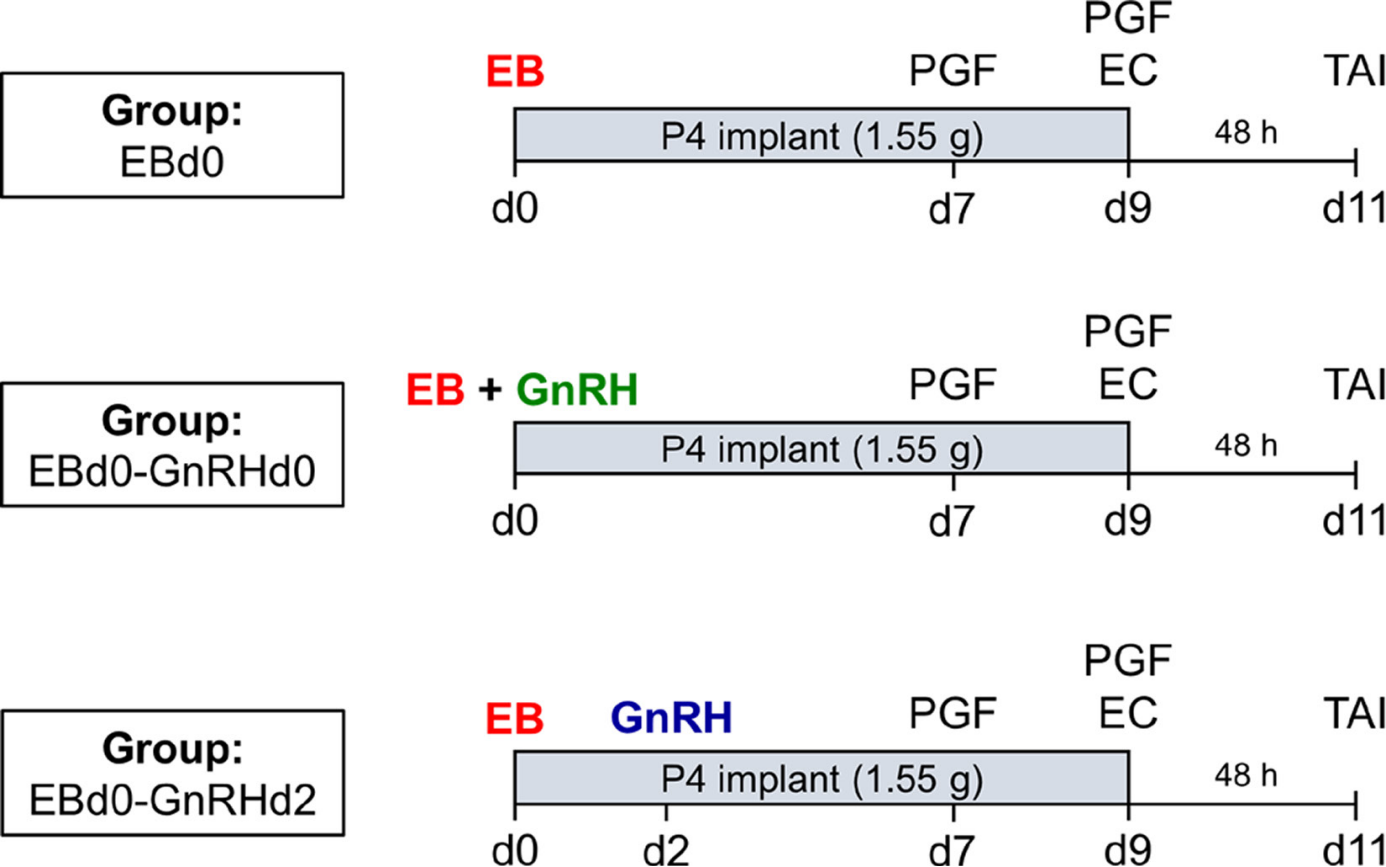
Experimental design with the hormonal treatments during timed AI (TAI) protocols. On d 0, all cows received a 1.55-g progesterone (P4) implant and, in the EBd0 group, cows received 2 mg of estradiol benzoate (EB). In the EBd0-GnRHd0 group, cows received 2 mg of EB plus 100 µg of gonadorelin diacetate tetrahydrate (GnRH) simultaneously on d 0, and in the EBd0-GnRHd2 group, cows received 2 of mg EB on d 0 and 100 µg of GnRH 48 h later, on d 2. The remaining treatments in the protocol were similar among groups, including 0.53 mg of cloprostenol sodium (PGF_2α_) on d 7, followed by a second PGF_2α_ on d 9, concomitant with P4 implant withdrawal and 1 mg of estradiol cypionate (EC). The TAI was performed on d 11 (48 h after P4 removal) in all experimental groups.

Statistical analyses were performed using SAS software (version 9.4 for Windows; SAS Institute Inc.). Analyses for continuous variables, such as DIM and milk production near TAI (7-d average production before TAI), were performed using the GLIMMIX procedure fitting a Gaussian distribution. Analyses of the binary response variable (P/AI on d 31) was performed using the GLIMMIX procedure, fitting a binomial distribution with the link logit function. Additionally, the option ddfm = kenwardroger was included in the model statement to adjust the degrees of freedom for variances.

The initial model for P/AI on d 31 included the effects of treatment, farm, parity (primiparous and multiparous), milk production class (< or ≥33.1 kg/d; Lopez et al., 2004), number of AI (first or later services), and the interactions between treatment and these variables. For the final model, only the interaction between farm and treatment was removed. To independently evaluate the effect of treatment in each class of cows within parity, milk production, and service number, the SLICE command was used in the GLIMMIX procedure.

The Tukey honestly significant difference post hoc test was performed to determine differences. Values are presented as least squares means (**LSM**) ± standard errors of the mean (**SEM**). Significant differences were declared when *P* < 0.05, whereas tendencies were considered when 0.10 > *P* ≥ 0.05.

The average DIM was 168.1 ± 4.1 and did not differ among treatments (*P* = 0.74) or between farms (*P* = 0.92). Similarly, milk production was not different among treatments (*P* = 0.64) or farms (*P* = 0.17), and multiparous cows had slightly greater milk production than primiparous cows (30.9 ± 0.4 vs. 29.1 ± 0.4 kg/d; *P* = 0.003).

Regarding P/AI on d 31, a treatment effect was detected (*P* = 0.04), in which cows in the EBd0-GnRHd2 group had greater fertility than EBd0 cows, whereas fertility of cows in the EBd0-GnRHd0 group did not differ from that in the other groups (Figure 2).

**Figure 2:**
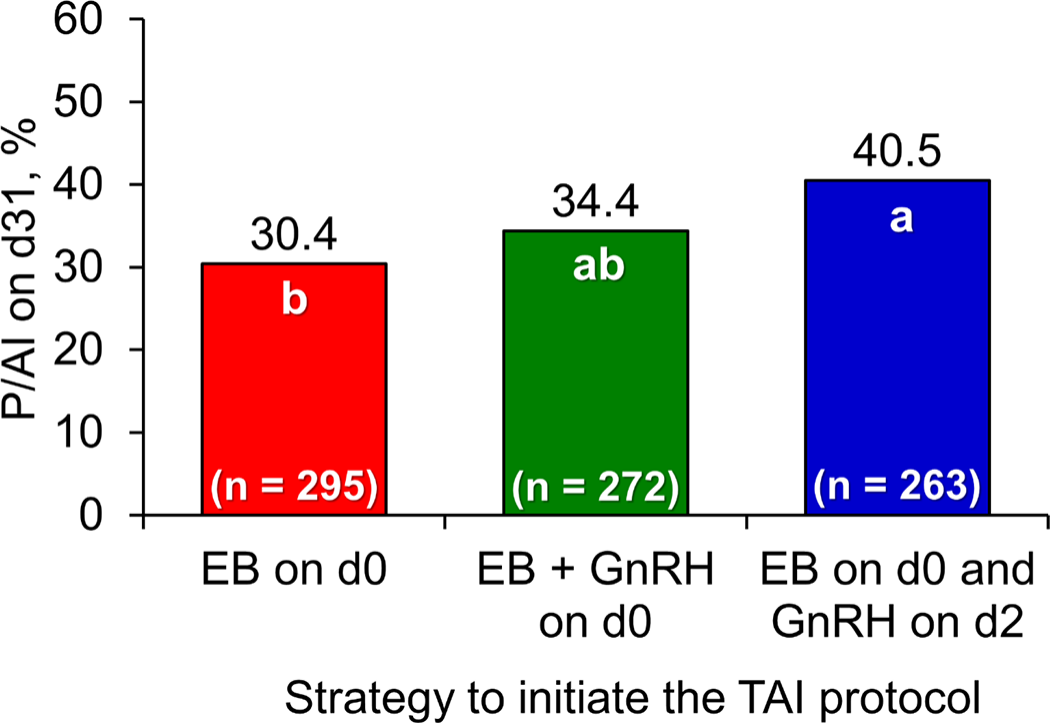
Pregnancy per AI (P/AI) 31 d after timed AI (TAI) according to the strategy to initiate the TAI protocol (*P* = 0.04). Treatments were estradiol benzoate (EB) on d 0, EB plus GnRH on d 0, or EB on d 0 and GnRH on d 2 of the TAI protocol. Means with different letters (a, b) are different (*P* < 0.05).

In a recent compilation of studies comprising 4,657 lactating dairy cows, Consentini et al. (2021) reported that the administration of only GnRH at the beginning of TAI protocols or its inclusion on d 0 or d 2 of an E2/P4-based protocol increased fertility by 17.9% compared with E2/P4-based protocols initiated only with EB (39.5 vs. 33.5%). Treatment with GnRH at the beginning of E2/P4-based protocols seems to increase fertility because of the induction of ovulation, which increases the proportion of cows with a functional CL at the time of treatment with PGF_2α_ and improves circulating P4 concentrations during the protocol (Pereira et al., 2015; Melo et al., 2016; Consentini et al., 2021). A study by Cerri et al. (2011) demonstrated that higher circulating P4 concentrations reduced LH pulse frequency during follicular development in a synchronization protocol, which is fundamental to ensure adequate growth of the dominant follicle in lactating dairy cows (Wiltbank et al., 2011a). Moreover, studies reported that higher circulating P4 concentrations during the protocol were associated with better embryo quality and greater fertility in dairy cows (Rivera et al., 2011; Wiltbank et al., 2011b).

In the present study, treatment with GnRH concomitant with EB on d 0 did not increase P/AI, in contrast to the results of Pereira et al. (2015), which reported a greater P/AI when GnRH was added on d 0 (30.7 vs. 26.8%). One reasonable explanation for the lack of effect on fertility with GnRH on d 0 may be the age of the ovulatory follicle at the end of the protocol. Because of the length of the protocol (11 d), cows from the EBd0-GnRHd0 group that ovulated to the GnRH given on d 0, although synchronized, may have had an older ovulatory follicle at the time of AI. Moreover, because of the 4-d period of proestrus (due to the first PGF_2α_ treatment on d 7), this follicle may have experienced overexposure to LH pulse frequency at the end of the protocol, compromising oocyte quality, which impairs fertility (Revah and Butler, 1996; Cerri et al., 2009; Monteiro et al., 2015). Conversely, results from the present study suggest that when cows ovulate to the GnRH treatment on d 2, the end of the protocol is similar to the traditional 5-d synchronization protocol, which results in a younger ovulatory follicle at the time of AI, resulting in greater P/AI. Indeed, according to Santos et al. (2010), the 5-d Cosynch72 with 2 PGF_2α_ treatments resulted in greater P/AI than the 7-d Cosynch72 with 1 PGF_2α_ treatment (37.9 vs. 30.9%). In addition, an interesting study comparing the 5-d Ovsynch protocol and the traditional Ovsynch, both with 2 PGF_2α_ treatments, reported similar fertility between these TAI programs (43.8 and 41.4%; Santos et al., 2016).

One aspect that could explain the lower fertility of the EBd0 group is the expected lack of emergence of a new follicular wave after EB plus P4 implant treatment in a percentage of cows (25–35%, Monteiro et al., 2015; Melo et al., 2018), resulting in low overall synchronization to the protocol in lactating dairy cows (32 to 60%; Monteiro et al., 2015). In this sense, the idea of adding a GnRH treatment on d 2 in the present study aimed to induce ovulation in cows that did not respond to the treatment with EB plus P4 implant, increasing the proportion of cows synchronized to the protocol. In addition, studies report that about 40% of cows with a CL on d 0 undergo CL regression during the synchronization protocol when treated with EB at the beginning of the synchronization protocol (Monteiro et al., 2015; Melo et al., 2016; Consentini et al., 2021), reducing circulating P4 concentrations during follicular development. These 2 situations can be partly overcome when a GnRH treatment is added at the beginning of the protocol.

Furthermore, we detected no effect of farm (*P* = 0.55) or interaction between farm and treatment (*P* = 0.92; Table 1). Likewise, number of AI had no effect on fertility (*P* = 0.25). Previous studies reported a marked decrease in P/AI as the number of services or DIM increased (Lopes et al., 2013). Although it is hard to draw conclusions on why the number of AI did not affect fertility in the current study, it is possible that a greater incidence of metabolic problems and more acute heat stress may have played an important role and could explain, in part, these contrasting results. Unsurprisingly, primiparous cows had greater P/AI than multiparous cows (*P* = 0.005; Table 1), as previously reported (Carvalho et al., 2014, 2015). This can be mainly explained by the lesser challenge related to liver steroid metabolism due to lower milk production and fewer health issues in the postpartum period in primiparous cows (Reinhardt et al., 2011; Pascottini et al., 2017).

**Table 1:** Pregnancy per AI (P/AI) 31 d after timed AI (TAI) according to the strategy to initiate the TAI protocol, farm, parity, milk production, and number of AI

Item	Overall	Strategy to initiate the TAI protocol^1^			*P*-value^2^		
		EBd0	EBd0-GnRHd0	EBd0-GnRHd2	T	V	I
Farm							
1	33.4 (137/398)	29.5 (55/161)	31.2 (40/125)	39.8 (42/112)	0.24	0.35	0.78
2	36.6 (156/432)	31.3 (43/134)	37.8 (54/147)	41.1 (59/151)	0.26		
Parity							
Primiparous	40.0 (149/371)^x^	35.3 (54/140)	43.2 (52/119)	41.7 (43/112)	0.46	0.005	0.27
Multiparous	30.3 (144/459)^y^	25.9 (44/155)^b^	26.6 (42/153)^b^	39.2 (58/151)^a^	0.03		
Milk production, kg/d							
<33.5	33.2 (187/540)	30.6 (65/190)	35.4 (64/178)	33.7 (58/172)	0.65	0.32	0.16
≥33.5	36.8 (106/290)	30.1 (33/105)^b^	33.5 (30/94)^b^	47.6 (43/91)^a^	0.04		
Number of AI							
First service	32.7 (98/294)	24.6 (21/86)^b^	34.1 (35/103)^ab^	40.6 (42/105)^a^	0.04	0.20	0.25
Later services	37.3 (195/536)	36.9 (77/209)	34.8 (59/169)	40.4 (59/158)	0.64		

a,b Least squares means with different superscripts within a row are different (*P* < 0.05).

x,y Least squares means with different superscripts within a column are different (*P* < 0.05) considering the main effect of the specific variable (farm, parity, milk production, and number of AI).

1 Treatments were estradiol benzoate (EB) on d 0 (EBd0), EB plus GnRH on d 0 (EBd0-GnRHd0), or EB on d 0 and GnRH on d 2 (EBd0-GnRHd2) of the TAI protocol.

2 T = effect of treatment within class of cows; V = main effect of the variable (farm, parity, milk production, and number of AI); and I = interaction between treatment and variable.

When additional analyses were performed to better understand the effect of treatment within specific classes of cows (Table 1), greater fertility was observed in cows with greater milk production (≥33.5 kg/d) in the EBd0-GnRHd2 group. This effect was also observed in multiparous cows and in cows receiving the first service (Table 1). Typically, these classes of cows have higher milk production (multiparous > primiparous, and first service > later services), which is closely related to a greater steroid hormone metabolic rate (Sangsritavong et al., 2002). This condition could compromise the emergence of a new follicular wave in response to EB plus P4 implant, in addition to reducing circulating P4 concentrations during follicular development, resulting in an older (and overexposed to LH) ovulatory follicle. Another possible explanation for the greater P/AI observed in these classes in the EBd0-GnRHd2 group, although not properly evaluated, is the expected greater incidence of cows in anovulatory condition, mainly in the first service (Monteiro et al., 2021), which would result in a greater number of cows without a CL at the beginning of the protocol. In both situations, addition of a GnRH treatment at the beginning of the TAI protocol could optimize synchronization and potentially improve fertility of lactating dairy cows. In the present study, this could partly explain the greater P/AI observed, especially in EBd0-GnRHd2 group compared with the EBd0 group.

Compliance and consistency of hormonal treatments is an important aspect when implementing synchronization protocols in dairy herds. The hormonal schedule must fit into the herd's weekly routine to make it as simple as possible. Thus, in addition to improving fertility, GnRH given on d 2 is ideal for the weekly routine of hormonal treatments, because it falls on the same day as P4 device removal in cows that started the synchronization protocol the week before. This is an important practical aspect because the additional GnRH on d 2 can be handled simultaneously with device removal in cows synchronized the previous week, making it easy to be implemented and ensuring good compliance while avoiding extra labor for managing cows during breeding routines.

In conclusion, addition of a GnRH treatment at the beginning of an E2/P4-based TAI protocol increased fertility only when GnRH was given on d 2. Moreover, the positive effect of this strategy was more pronounced in multiparous cows, cows with greater milk production, and cows in the first service, which could have benefited more from better synchronization, higher circulating P4 concentrations during the protocol, and a younger (and not overexposed to LH) ovulatory follicle at the end of the protocol.
